# Uterine adenocarcinoma with peritoneal carcinomatosis in a cat: HBME-1 as a potential marker for uterine epithelial disorders in queens

**DOI:** 10.1007/s11259-026-11191-7

**Published:** 2026-04-07

**Authors:** Gabriela Hartmann, Éryca Ceolin Lamego, Milena Carolina Paz, Marcele Bettim Bandinelli, Saulo Petinatti Pavarini

**Affiliations:** Setor de Patologia Veterinária, Universidade Federal do Rio Grande do Sul (UFRGS), Porto Alegre, Rio Grande do Sul Brazil

**Keywords:** Endometrial adenocarcinoma, Uterine neoplasia, Genital system neoplasia, Progesterone receptor, Estrogen receptor alpha

## Abstract

Uterine adenocarcinomas and the spread of the endometrial neoplastic cells throughout the abdominal cavity (peritoneal carcinomatosis) are rare in cats. The Hector Battifora Mesothelial Epitope-1 (HBME-1) is a widely used monoclonal antibody in human medicine, including uterine disorders, but is scarcely explored in veterinary medicine. Herein, we describe a case of uterine adenocarcinoma with peritoneal carcinomatosis and HBME-1 immunolabeling of the neoplastic cells in a female cat. Clinically, the cat was admitted to a veterinary teaching hospital with fever, and was submitted to ovariohysterectomy after a suspicion of pyometra. The uterus was sent for histopathological analysis, and a diagnosis of uterine adenocarcinoma was made. The animal was euthanized three months after the initial diagnosis, and the cadaver was sent for postmortem examination. At necropsy, there was an irregular-shaped, firm, white, 5 × 3 × 2.5 cm mass in the uterine stump, and multiple 0.3 to 0.7 cm diameter nodules of similar characteristics within the peritoneal cavity. Histologically, the tumors consisted of an infiltrative neoplastic proliferation of epithelial cells arranged in acini and papillae, accompanied by an abundant desmoplastic reaction and marked nuclear and cellular pleomorphism. By immunohistochemistry, the neoplastic cells were reactive for HBME-1, pancytokeratin, progesterone receptor, and estrogen receptor alpha. Additionally, the normal endometrium exhibited apical immunolabeling for HMBE-1. To our knowledge, HBME-1 labeling has never been reported in normal or neoplastic endometrial cells in domestic species. Our findings may expand the application of this antibody in veterinary pathology and broaden the immunohistochemical panel of uterine adenocarcinomas in cats.

**Supplementary Information:**

The online version contains supplementary material available at 10.1007/s11259-026-11191-7.

## Background

Tumors of the urogenital tract are rare in cats (Sapierzyński et al. 2007). Within this system, the uterus is the most common site of neoplasia; however, it accounts for only 0.29% of all feline neoplasia (Miller et al. 2003). Among those uterine neoplasms, epithelial tumors, mostly uterine adenocarcinomas, are considered rare (Kennedy et al. 1998; Agnew and MacLachlan 2017). Besides that, a few case series have been reported, suggesting that these neoplasms may be more common than once considered (Papparella and Roperto 1984; Miller et al. 2003; Gil da Costa et al. 2009; Sontas et al. 2013; Saraiva et al. 2015). Rarely, these neoplasms are reported to disseminate throughout the abdominal cavity as peritoneal carcinomatosis (Miller et al. 2003). On immunohistochemical panels, feline uterine adenocarcinomas typically demonstrate positive immunolabeling for cytokeratins, estrogen receptor alpha (ERα), and progesterone receptor (PR) (Miller et al. 2003; Gil da Costa et al. 2009; Saraiva et al. 2015).

The Hector Battifora Mesothelial Epitope-1 (HBME-1) is a monoclonal antibody that reacts with an antigen of mesothelial cells’ microvilli (Bacci et al. 2006). In veterinary medicine literature, this antibody was tested and optimized only for use in canine ovarian tumors (Banco et al. 2011) and feline and canine mesotheliomas (Bacci et al. 2006; Vascellari et al. 2011). In contrast, this antibody is broadly used in human pathology (Cazzaniga et al. 2022), including gynecopathology, where the immunolabeling by this molecule is observed in the normal endocervical cells (Hao et al. 2010), and in neoplastic cells of benign and malignant conditions, such as uterine adenomatoid tumors and uterine adenocarcinomas, respectively (Hao et al. 2010; Chen et al. 2017). Therefore, this paper aims to describe the clinical, gross, histopathological, and immunohistochemical findings in a rare case of uterine adenocarcinoma with carcinomatosis in a cat, with HBME-1 labeling within the neoplastic cells of the uterine neoplasm, peritoneal metastasis, and normal endometrial cells.

## Case presentation

A 12-year-old female domestic shorthair cat was brought to a veterinary teaching hospital with fever and serous to purulent vaginal discharge. After a clinical diagnosis of pyometra, the cat was ovariohysterectomized. The removed uterus was sent for histopathological analysis, which revealed an endometrial adenocarcinoma within the uterus. After the diagnosis, an abdominal radiography was performed to investigate possible remaining uterine nodules or abdominal metastases, which were absent. Three months after the surgery, the cat presented with dysuria. The cat’s owners returned to veterinary care, and after an abdominal ultrasonographic exam, abdominal masses were confirmed in the urinary bladder region. Three days after the exam, the patient had severe dyschezia and dysuria and was hospitalized for urinary catheterization. Following several unsuccessful attempts at catheterization, the cat was euthanized two days after hospitalization. The cat was sent for post-mortem examination at the Veterinary Pathology Section of the Universidade Federal do Rio Grande do Sul.

**Fig. 1:**
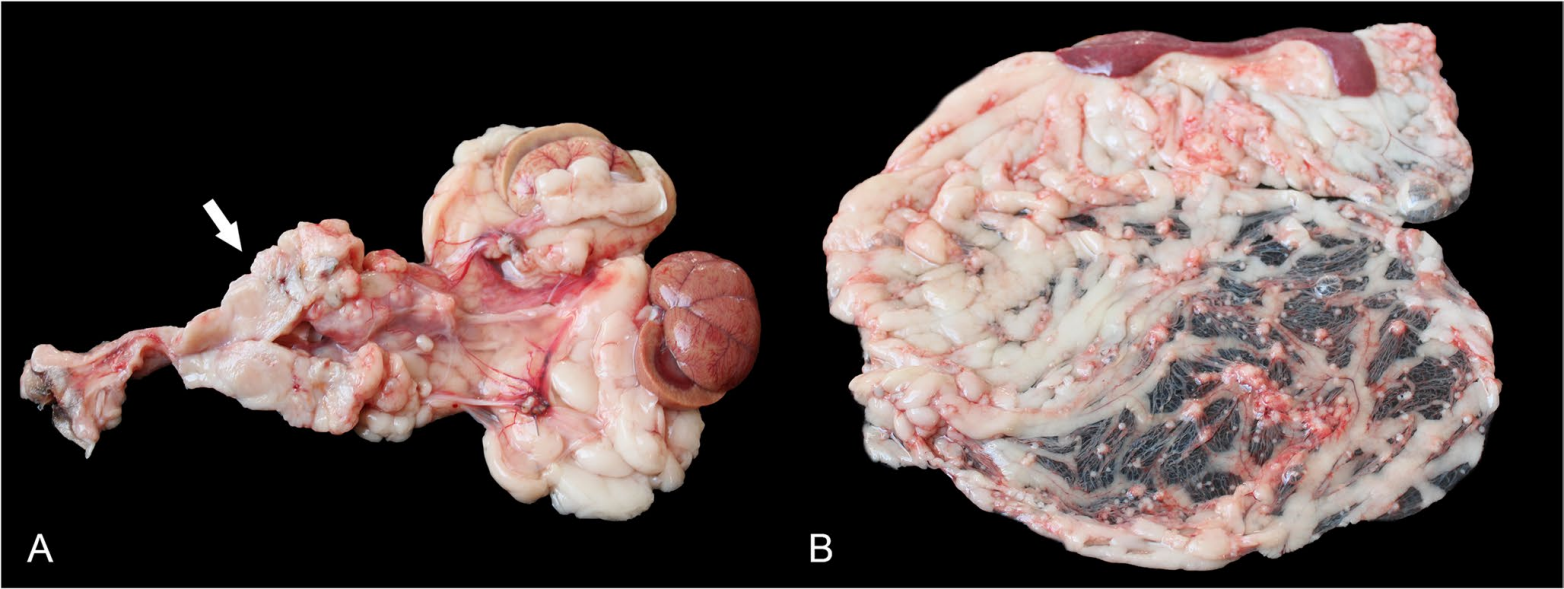
Gross features of a case of uterine adenocarcinoma with carcinomatosis in a cat. **A** Cut surface of an irregular-shaped, 5 × 3 × 2.5 cm, white, firm, multilobulated mass (arrow) in the uterine stump site. **B** Peritoneal carcinomatosis. Multifocal to coalescing 0.3 to 0.7 cm irregular, white nodules throughout the omentum

At necropsy, there was about 60 mL of red translucent fluid in the abdominal cavity. In the uterine stump, there was an irregular-shaped, firm, diffusely white, multilobulated, 5 × 3 × 2.5 cm mass that ventrally compressed the urethra and dorsally, the rectum **(**Fig. 1A**)**. The urethral wall was diffusely thickened, firm, and homogeneously white. In the kidneys, the renal pelvis was moderately dilated and filled with urine (hydronephrosis). Additionally, in the omentum, mesenterium, diaphragm, parietal peritoneum, rectus abdominis muscle, and the formerly ovarian sites, there were multifocal to coalescing, white, firm white nodules of 0.3 to 0.7 cm diameter **(**Fig. 1B**)**. No gross lesions were observed in the mammary glands. Sections of the uterine stump, urinary bladder, urethra, kidneys, small and large intestines, stomach, pancreas, liver, spleen, adrenal glands, bone marrow, lungs, heart, thyroid glands, and encephalon were collected and fixed in 10% neutral-buffered formalin for 48 h.

After fixation, the fragments underwent routine processing and were embedded in paraffin for histopathological evaluation. Slides with 3 μm-thick sections were stained with hematoxylin and eosin (HE). Additionally, 2 μm-thick, deparaffinized, rehydrated, formalin-fixed, paraffin-embedded selected tissue sections of the first uterine biopsy specimen, uterine stump mass, and abdominal carcinomatosis were submitted to anti-human pancytokeratin, anti-human vimentin, anti-ERα, anti-PR, and anti-HBME-1 immunohistochemical assays. Details of the antibodies, dilutions, antigen retrieval methods, and positive controls are listed in Table 1. The primary antibodies were diluted in commercial antibody diluent (Dako, www.agilent.com) and incubated overnight for 12 h at room temperature (20 °C). NovoLink Max Polymer Detection System (Leica Biosystems, www.leicabiosystems.com) was used as the detection system. The slides were counterstained with Harris’ hematoxylin. As a negative control, Negative Control Serum (Biocare Medical) was used instead of the primary antibodies.

**Table 1:** Antibodies and immunohistochemical protocols used in a case of uterine adenocarcinoma with carcinomatosis in a cat

Antibody	Dilution	Antigen retrieval	Chromogen	Positive control
Anti-pancytokeratin, monoclonal (clone AE1/AE2)^a^	Ready-to-use	40 min at 96 °C, digital pressure cooker, citrate buffer (pH 6.0)	AEC^f^	Normal feline skin
Anti-vimentin, monoclonal (clone V9)^b^	1:200	40 min at 96 °C, digital pressure cooker, citrate buffer (pH 6.0)	AEC	Normal feline skin
Anti-ERα (clone 6F11)^c^	Ready-to-use	15 min at 125 °C, digital pressure cooker, Tris-EDTA buffer (pH 9.0)	AEC	Normal feline mammary gland
Anti-PR (clone 1A6)^d^	1:50	20 min at 125 °C, digital pressure cooker, citrate buffer (pH 6.0)	DAB^a^	Normal feline mammary gland
Anti-HBME-1, monoclonal (clone HBME-1)^e^	1:100	30 min at 97 °C, digital pressure cooker, Tris-EDTA buffer (pH 9.0)	AEC	Canine ovary

*AEC* 3-Amino-9-Ethylcarbazole, *DAB* 3,3’-Diaminobenzidine, *EDTA* Ethylenediaminetetraacetic Acid; *ERα* estrogen receptor alpha, *PR* progesterone receptor, *HBME-1* Hector Battifora Mesothelial Epitope

^a^Dako, Carpinteria, California, USA

^b^Zymed, Thermo Fischer Scientific, Massachusetts, USA

^c^EasyPath, Erviegas, São Paulo, BR

^d^Leica Biosystems, Newcastle, UK

^e^Cell Marque, California, USA

^f^Biocare Medical, California, USA

**Fig. 2:**
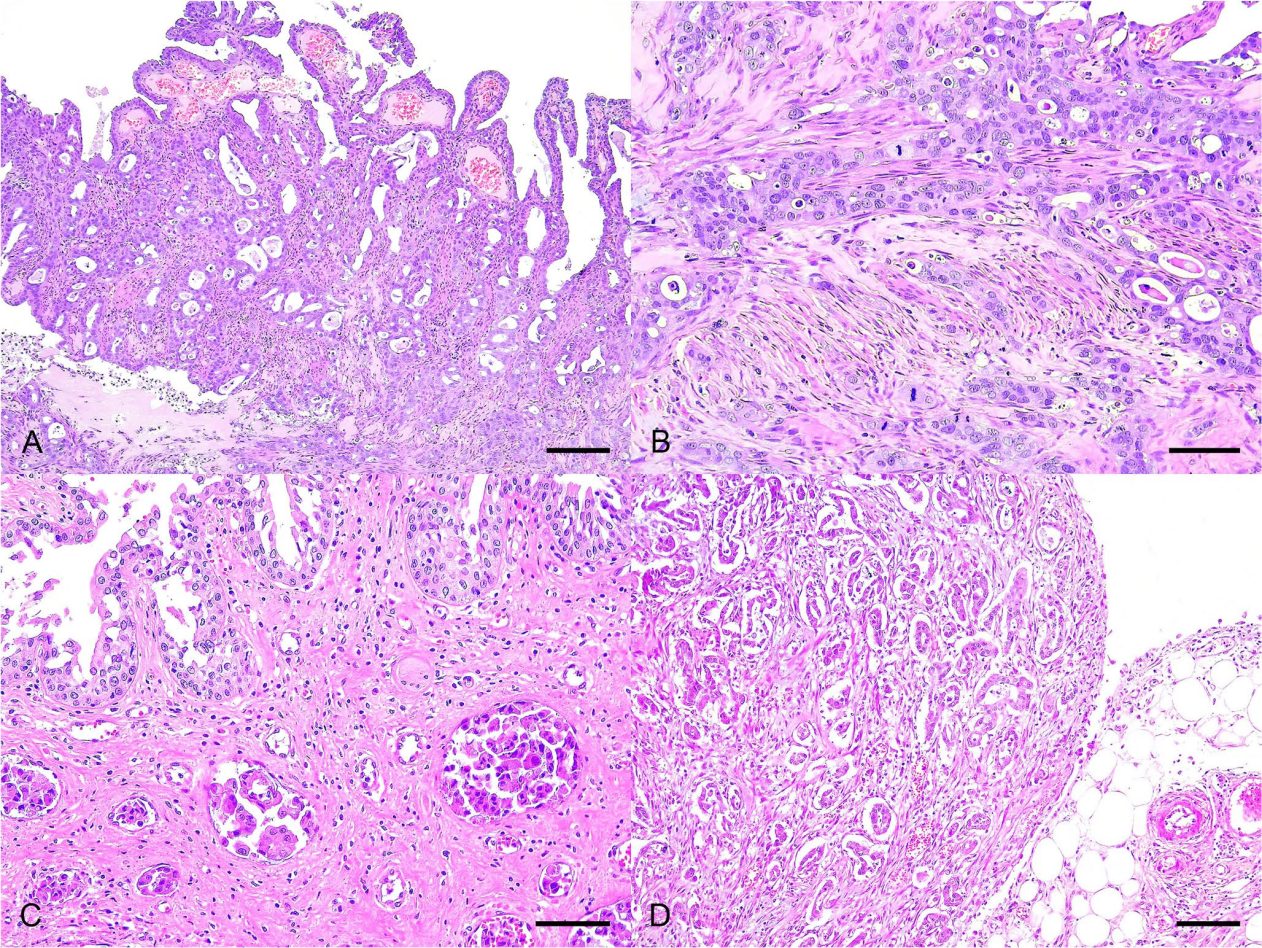
Histopathological features of a case of uterine adenocarcinoma with carcinomatosis in a cat. **A** Primary uterine neoplasm. Papillary and acinar arrangement of the neoplastic cells. Hematoxylin and eosin (H&E). Bar = 300 μm. **B** Uterine stump metastasis. The neoplastic cells were columnar to polyhedral, had a lightly eosinophilic cytoplasm with indistinct cell borders, round to oval nuclei with coarse to clumped chromatin, and 1–2 nucleoli. There were marked cellular and nuclear pleomorphism, and mitotic figures were frequent. H&E. Bar = 200 μm. **C** Urinary bladder metastasis. Multiple neoplastic emboli within the submucosal vessels A mild, multifocal macrophage infiltrate is seen within the lamina propria/submucosa. H&E. Bar = 150 μm. **D** Peritoneal carcinomatosis. Metastasis within the omentum composed of multiple neoplastic cell acini and intense desmoplastic reaction. H&E. Bar = 300 μm

Histologically, the primary uterine neoplasm and the uterine stump masses were composed of a transmural, poorly circumscribed, infiltrative neoplastic proliferation of epithelial cells arranged in acini and papillae, sustained by a fine fibrovascular stroma **(**Fig. 2A**)**. The cells were collunar to polyhedral, with abundant eosinophilic cytoplasm of indistinct cell borders, round to oval nuclei, coarse to clumped chromatin, and 1–2 conspicuous nucleoli **(**Fig. 2B**)**. The neoplastic cells had marked anisocystosis and anisokaryosis. Numerous neoplastic cells exhibited karyomegaly and macronucleoli, and nuclear molding, cannibalism, binucleation, and multinucleation were frequently observed. There were 31 mitotic figures in a 2.37 mm² area (10 FN22/x40 fields), with lymphovascular space invasion (LVSI). The acinar lumen was often filled with amorphous to fibrilar, lightly basophilic material, intact and degenerate neutrophils, cellular debris, and plumped macrophages with vacuolated cytoplasm. Interpersed with the neoplasm, there was marked fibrous connective tissue deposition (desmoplastic reaction), and multifocal mild inflammatory infiltrate composed of intact and degenerate neutrophils and macrophages. In the urethra and urinary bladder neck, there was a similar, transmural, desmoplastic neoplastic proliferation with lymphovascular invasion **(**Fig. 2C**)**. The same neoplastic proliferation was seen in the omentum **(**Fig. 2D**)**, mesenterium, diaphragm, parietal peritoneum, rectus abdominis muscle, and the adipose tissue of the formerly ovarian sites. Additionally, in the small intestine, there was a diffuse epitheliotropic neoplastic proliferation composed of small lymphocytes within the lamina propria.

**Fig. 3:**
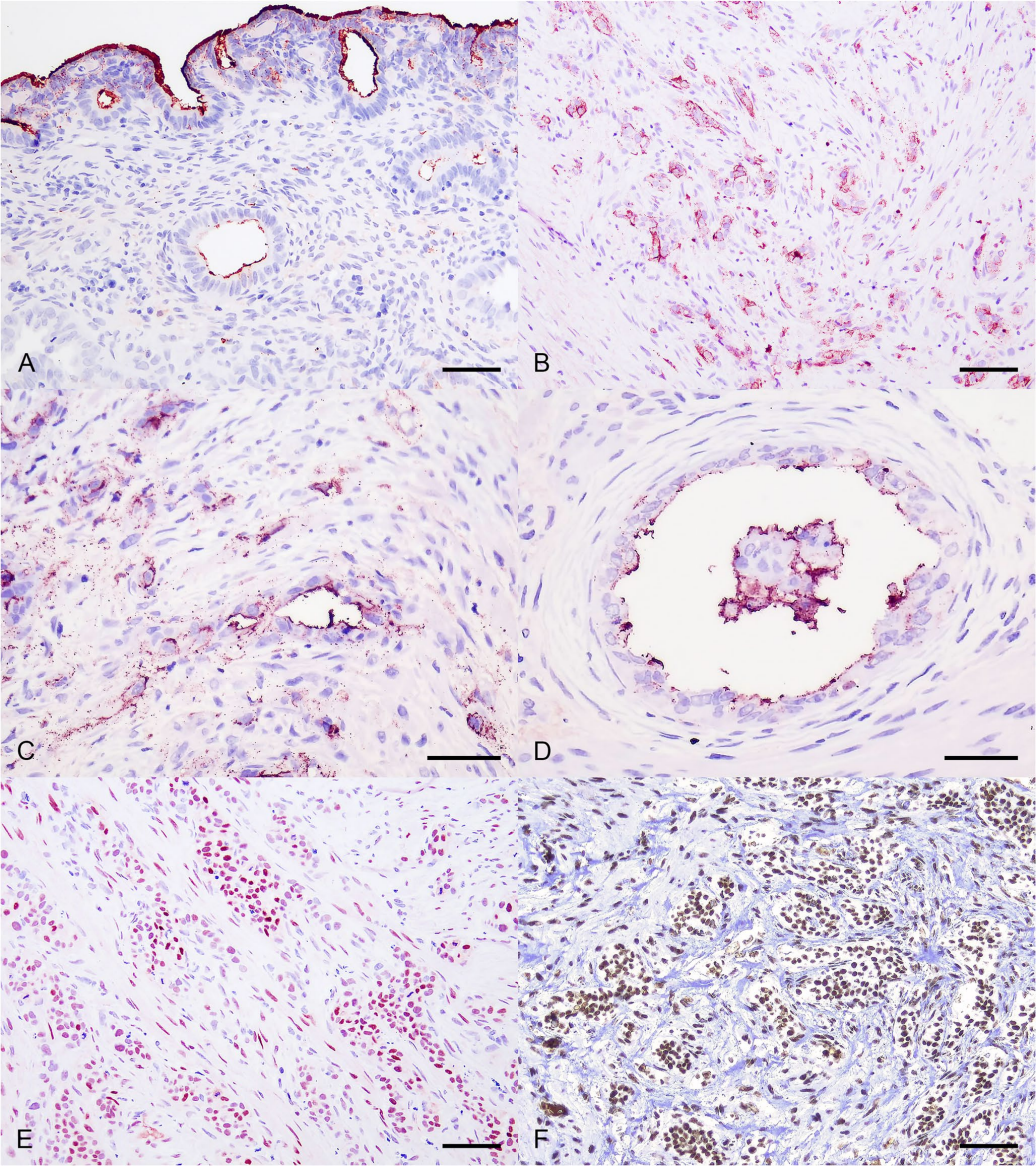
Immunohistochemical features of a case of uterine adenocarcinoma with carcinomatosis in a cat. **A** The normal superficial epithelium and epithelium of the endometrial glands showed diffuse, apical immunolabeling for HBME-1. Immunohistochemistry (IHC). AEC chromogen. Bar = 200 μm. **B** Apical/membranous labeling pattern of HBME-1 within the uterine stump neoplastic cells. IHC. AEC chromogen. Bar = 200 μm. **C **and** D.** Close-up view of the apical immunolabeling for HBME-1 at the neoplastic cells. IHC. AEC chromogen. Bar = 100 μm. **E.** Diffuse nuclear immunolabeling for estrogen receptor alpha in the uterine neoplastic cells. IHC. AEC chromogen. Bar = 200 μm. **F.** Diffuse nuclear immunolabeling for progesterone receptor in the uterine neoplastic cells. IHC. DAB chromogen. Bar = 200 μm

In the main uterine neoplasm, uterine stump, and peritoneal metastases, 100% of the neoplastic cells showed cytoplasmic/membranous immunolabeling for pancytokeratin. Regarding HBME-1 immunolabeling, approximately 80% of the neoplastic cells in all three sites exhibited apical/membranous immunolabeling; however, the staining intensity was more pronounced in the main uterine neoplasm. In addition to the neoplastic cells, over 95% of normal endometrial superficial epithelium and the epithelium of the endometrial glands also showed apical HBME-1 labeling **(**Figs. 3A-B**)**. About 95% of the neoplastic cells in the main uterine neoplasm demonstrated nuclear immunolabeling for ERα **(**Fig. 3C**)**. In contrast, ERα labeling was absent in the neoplastic cells of both the uterine stump and peritoneal metastases. Furthermore, approximately 98% of the neoplastic cells in the primary uterine neoplasm, as well as the myometrial cells, displayed nuclear PR immunolabeling **(**Fig. 3D**)**. In contrast, only about 30% of the neoplastic cells in the uterine stump and 50% of those in the peritoneal metastases exhibited PR immunolabeling, which was frequently observed as cytoplasmic labeling. In the uterine stump, decreased nuclear PR immunolabeling was also noted in the myometrial cells. Loss of PR nuclear labeling was most evident in exfoliated luminal neoplastic cells, which showed loss of intercellular cohesion. The neoplastic cells of the main uterine tumor, uterine stump, and peritoneal metastases showed absent vimentin immunolabeling.

## Discussion

A diagnosis of uterine adenocarcinoma with abdominal carcinomatosis was established based on the gross, histopathological, and immunohistochemical findings in this case. Although rare in cats and dogs (Agnew and MacLachlan 2017; De Sousa et al. 2025), uterine adenocarcinomas are relatively common incidental findings in aged cows at slaughter (Kennedy et al. 1998; Lucena et al. 2011; Agnew and MacLachlan 2017) and represent the most frequent spontaneous tumor in female rabbits (Baum 2021; Mäkitaipale et al. 2022). In women, endocervical adenocarcinoma is the second most common tumor of the uterine cervix, surpassed only by cervical squamous cell carcinoma (Smith et al. 2000; Hodgson et al. 2019). Both neoplasms are strongly associated with human papillomavirus (HPV) infection (Pirog 2017; Hodgson et al. 2019). In cats, a possible viral association has been suggested in a report describing a uterine adenocarcinoma in a feline leukemia virus (FeLV)-positive two-year-old cat (Cho et al. 2011). However, this link is unlikely, since similar neoplasms have been reported in kittens as young as one year old (Payan-Carreira et al. 2013; Sontas et al. 2013). Uterine adenocarcinomas are more frequent in mature adult and senior queens, as observed in the present case (Miller et al. 2003; Sapierzyński et al. 2007; Anderson and Pratschke 2011; Dornbusch et al. 2020). In our case, the initial clinical suspicion was pyometra, which prompted surgical removal of the uterus. Uterine adenocarcinomas can be clinically misdiagnosed as pyometra in older queens, as both conditions may occur simultaneously (Dornbusch et al. 2020). Therefore, submission of the uterus for histopathological examination is essential in all cases of suspected pyometra in queens.

In our case, peritoneal carcinomatosis and local invasion of the urinary bladder/urethra were present; however, no distant metastases were observed, although lung and liver metastases are reported in cats (Agnew and MacLachlan 2017). In women, metastatic spread most frequently involves pelvic and para-aortic lymph nodes, with less common dissemination to the lungs and liver (Creasman et al. 2006; Kurman et al. 2014). Urinary bladder invasion, as observed in our case, is a feature of high-grade uterine adenocarcinomas in women (FIGO stage IV) (Creasman et al. 2006; Berek et al. 2023). Regarding LVSI, as observed in this case, it is a common feature in endometrial adenocarcinomas in women, considered an adverse prognostic factor associated with an increased risk of regional lymph node involvement and distant metastasis (Bosse et al. 2015). In cattle and rabbits, lymphovascular invasion and distant metastasis are reported less frequently; when present, metastatic sites most commonly include regional lymph nodes, liver, and lungs (Kennedy et al. 1998; Percy and Barthold 2007; Stilwell and Peleteiro, 2010; Lucena et al. 2011; Varga 2013; Agnew and MacLachlan 2017).

In the present case, myometrial invasion was likely already present at the time of uterine removal, in accordance with the well-described pattern whereby uterine carcinomas arise from the endometrium and progress into the myometrium (Agnew and MacLachlan 2017). This invasion was evidenced by the presence of multiple neoplastic acini interspersed among myocytes in the uterine stump on HE-stained slides. However, in some instances, uterine adenocarcinomas can arise de novo from the uterine stump in ovariohysterectomized queens (Anderson et al. 2011). Carcinomatosis refers to the widespread dissemination of malignant epithelial cells throughout the body cavities (Weston et al. 2021). In cats, carcinomas originating from the pancreas, liver, or small intestine have the highest propensity to metastasize to the peritoneum, resulting in peritoneal carcinomatosis (Monteiro and O'Brien, 2004 ; Weston et al. 2021; Cony et al. 2023). In women and cattle, peritoneal carcinomatosis is often associated with uterine adenocarcinomas, particularly in high-grade or advanced-stage tumors (Lee et al. 2003; Creasman et al. 2006; Stilwell and Peleteiro 2010; Berek et al. 2023); however, such an association has been rarely reported in cats (Miller et al. 2003). In the present case, peritoneal dissemination of the uterine neoplasm was observed after uterine removal, highlighting the malignant potential of these tumors in cats.

In cats, uterine adenocarcinomas typically exhibit cytoplasmic or membrane-associated cytokeratin and nuclear PR immunolabeling (Miller et al. 2003; Gil da Costa et al. 2009). These features were observed in the neoplastic cells of the uterus, uterine stump, and peritoneal carcinomatosis in our case. However, PR labeling was more pronounced in the neoplastic cells of the primary uterine tumor than in the metastatic lesions. Regarding ERα, immunolabeling is detected in approximately half of reported cases of uterine adenocarcinomas in cats (Miller et al. 2003). In the present case, the primary uterine neoplasm exhibited diffuse nuclear immunolabeling for ERα, whereas it was absent in the neoplastic cells of the uterine stump and peritoneal metastases. This was an expected finding since the loss of ERα labeling in feline uterine adenocarcinomas is generally more pronounced than the loss of PR immunolabeling compared to normal endometrial cells (Saraiva et al. 2015). Moreover, the reduction or loss of ERα and PR labeling in metastatic sites is also a common finding in women with endometrioid endometrial carcinoma (Bartosch et al. 2015).

However, it is also important to consider, in this case, that tissue autolysis may have influenced these results on ER and PR immunolabeling. The primary uterine neoplasm was submitted as a biopsy specimen to the veterinary pathology laboratory, whereas the uterine stump and peritoneal metastases were collected postmortem. Consequently, autolysis was more advanced in the latter samples. As previously reported, a delay in tissue fixation can negatively affect hormone receptor immunolabeling, including PR and ERα, as demonstrated in human breast cancer samples (Oyama et al. 2007; Yildiz-Aktas et al. 2012). The fact that the normal myometrial cells surrounding the neoplastic cells have also lost PR labeling strengthens this hypothesis of a delayed fixation artifact in our case. Therefore, this case highlights the importance of controlling the cold ischemic time period before fixation in samples in which hormonal markers will be used—such as mammary and uterine tumors—since a cold ischemic time as short as one-half hour may lead to a significant reduction in IHC staining, especially for PR (Yildiz-Aktas et al. 2012).

Besides that, the most noteworthy finding in our case was the strong HBME-1 immunolabeling observed in the neoplastic cells across all analyzed samples. HBME-1 is a widely used antibody in human medicine, primarily applied in the detection of mesothelial and thyroid malignancies (Cazzaniga et al. 2022). Moreover, in women, HBME-1 immunolabeling is reported in the normal endocervix, endocervicosis, endometrial glands, endocervical adenocarcinomas, and uterine adenomatoid tumors (Hao et al. 2010; Nakaguro et al. 2016; Chen et al. 2017; Cazzaniga et al. 2022). In our case, HBME-1 labeling was also detected in normal endometrial cells, suggesting that this antibody could serve as a potential marker for uterine epithelial neoplasia and other endometrial disorders in queens, as it is already widely applied in human gynecopathology. However, it is important to note that HBME-1 should not be used as a sole immunomarker to confirm endometrial origin in cases of peritoneal carcinomatosis in hysterectomized queens, since mesotheliomas in cats may also exhibit HBME-1 immunolabeling (Bacci et al. 2006). In these cases, in addition to the gross and histopathological findings, ERα and PR assist in the confirmation of the uterine adenocarcinoma diagnosis in cases with carcinomatosis.

In veterinary pathology, only a few studies were published on HBME-1 immunolabeling in domestic species (Bacci et al. 2006; Banco et al. 2011; Vascellari et al. 2011). HBME-1 labeling is observed in normal epithelial ovarian surface and epithelial ovarian tumors in dogs (Banco et al. 2011) and in mesotheliomas in cats and dogs (Bacci et al. 2006; Vascellari et al. 2011). To the authors’ knowledge, HBME-1 immunolabeling has never been published in a case of uterine adenocarcinoma or normal endometrium in a domestic species. This apical/membranous labeling pattern of HBME-1 observed in this case is one of the reported immunolabeling patterns of this antibody in human tissues (Cazzaniga et al. 2022). The same pattern was observed in ovarian epithelial neoplastic cells in dogs (Banco et al. 2011) and mesothelial neoplastic cells in cats and dogs (Bacci et al. 2006; Vascellari et al. 2011). Additionally, in human tissues, normal and neoplastic epithelial cells of the lungs, normal and neoplastic cartilage, myoepithelial cells, ductal carcinomas of the mammary gland, and thyroid carcinomas exhibit HBME-1 immunolabeling (Cazzaniga et al. 2022). Therefore, HBME-1 is a pluripotential marker that could be further explored in veterinary pathology.

This report highlights a rare case of uterine adenocarcinoma with carcinomatosis in a 12-year-old queen, emphasising the diagnostic significance of gross, histopathological, and immunohistochemical findings. The identification of HBME-1 immunolabeling in both the primary uterine, uterine stump, and peritoneal carcinomatosis neoplastic cells is a novel finding in feline pathology, expanding the immunohistochemical profile of this rare neoplasm. Additionally, the immunolabeling by this antibody in normal endometrial cells suggests that HBME-1 may also be employed in other endometrial disorders of queens. Further studies may elucidate this hypothesis. Given the established role of HBME-1 in human pathology and limited veterinary application, this marker shows promise for broader diagnostic use in domestic animals. Moreover, our study reinforces the need for further research on cold ischemic time and the immunolabeling for hormonal markers in veterinary pathology.

## Supplementary Information

Below is the link to the electronic supplementary material.

Supplementary Material 1.

## Data Availability

No datasets were generated or analysed during the current study.
