# Supplementary figures and images for: Uterine adenocarcinoma with peritoneal carcinomatosis in a cat: HBME-1 as a potential marker for uterine epithelial disorders in queens
Source: Vet Res Commun. 2026 Apr 7;50(4):252. doi: 10.1007/s11259-026-11191-7 (PMC13056768; doi:10.1007/s11259-026-11191-7)

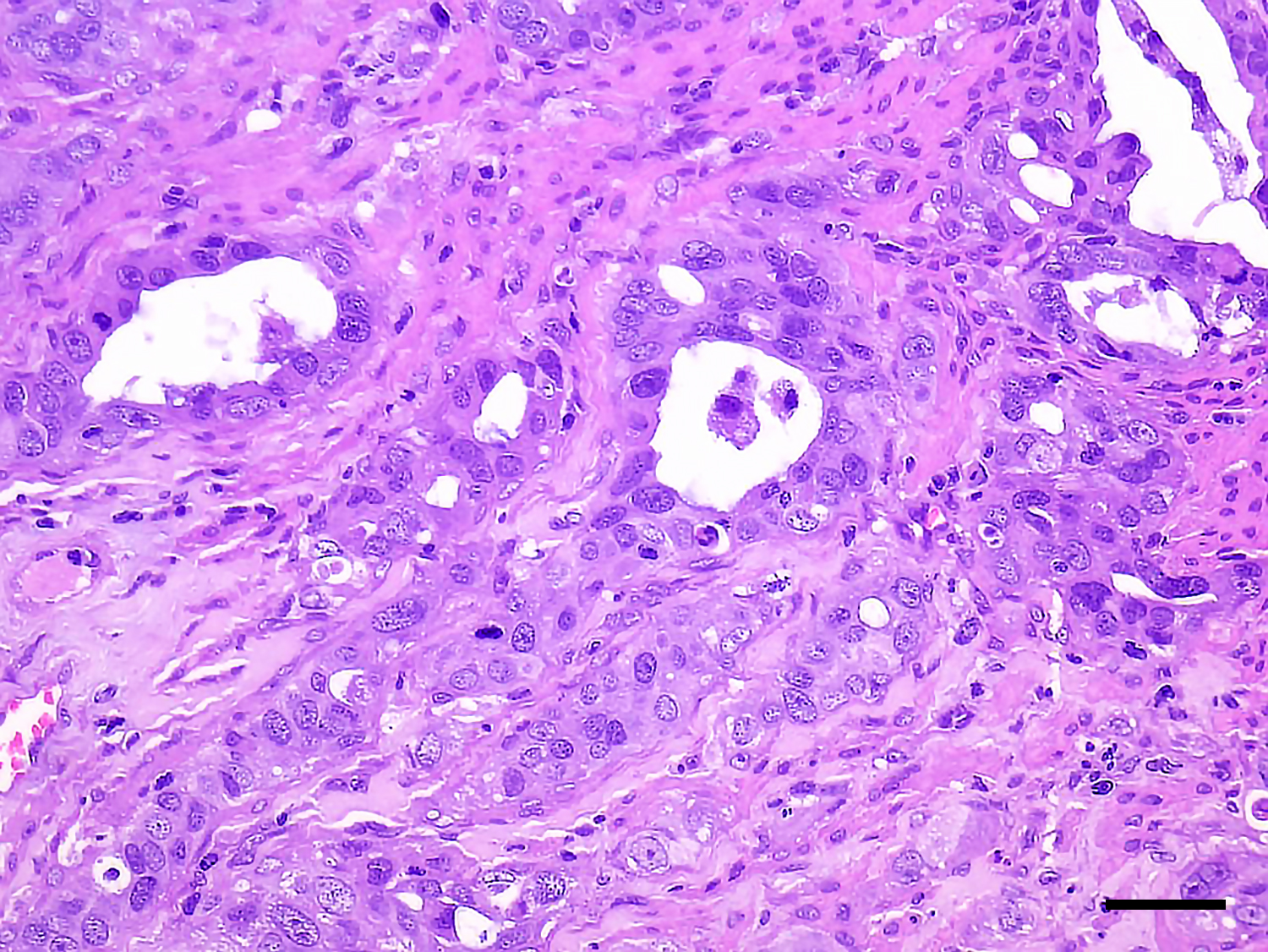

Supplement: Supplementary file 1 — Supplementary Material 1. [file 11259_2026_11191_MOESM1_ESM.tif]
